# Discovery and replication of microRNAs for breast cancer risk using genome-wide profiling

**DOI:** 10.18632/oncotarget.13241

**Published:** 2016-11-09

**Authors:** Cenny Taslim, Daniel Y. Weng, Theodore M. Brasky, Ramona G. Dumitrescu, Kun Huang, Bhaskar V.S. Kallakury, Shiva Krishnan, Adana A. Llanos, Catalin Marian, Joseph McElroy, Sallie S. Schneider, Scott L. Spear, Melissa A. Troester, Jo L. Freudenheim, Susan Geyer, Peter G. Shields

**Affiliations:** ^1^ Comprehensive Cancer Center, The Ohio State University, Columbus, OH, USA; ^2^ Distilled Spirits Council of the United States, Washington, DC, USA; ^3^ Department of Pathology, Georgetown University, Washington, DC, USA; ^4^ Department of Epidemiology, Rutgers University, New Brunswick, NJ, USA; ^5^ Center for Biostatistics, Comprehensive Cancer Center, The Ohio State University, Columbus, OH, USA; ^6^ Pioneer Valley Life Sciences Institute, Springfield, MA, USA; ^7^ Department of Plastic Surgery, Georgetown University Hospital, Washington, DC, USA; ^8^ Lineberger Comprehensive Cancer Center, University of North Carolina at Chapel Hill, Chapel Hill, NC, USA; ^9^ Departement of Epidemiology and Environmental Health, School of Public Health and Health Professions, University at Buffalo, Buffalo, NY, USA; ^10^ Health Informatics Institute, University of South Florida, Tampa, FL, USA; ^11^ Victor Babes University of Medicine and Pharmacy, Timisoara, Romania

**Keywords:** microRNA, epigenetics, breast cancer risk prediction, tissue-based biomarkers, healthy women

## Abstract

**Background:**

Genome-wide miRNA expression may be useful for predicting breast cancer risk and/or for the early detection of breast cancer.

**Results:**

A 41-miRNA model distinguished breast cancer risk in the discovery study (accuracy of 83.3%), which was replicated in the independent study (accuracy = 63.4%, P=0.09). Among the 41 miRNA, 20 miRNAs were detectable in serum, and predicted breast cancer occurrence within 18 months of blood draw (accuracy 53%, *P*=0.06). These risk-related miRNAs were enriched for HER-2 and estrogen-dependent breast cancer signaling.

**Materials and Methods:**

MiRNAs were assessed in two cross-sectional studies of women without breast cancer and a nested case-control study of breast cancer. Using breast tissues, a multivariate analysis was used to model women with high and low breast cancer risk (based upon Gail risk model) in a discovery study of women without breast cancer (*n*=90), and applied to an independent replication study (*n*=71). The model was then assessed using serum samples from the nested case-control study (*n*=410).

**Conclusions:**

Studying breast tissues of women without breast cancer revealed miRNAs correlated with breast cancer risk, which were then found to be altered in the serum of women who later developed breast cancer. These results serve as proof-of-principle that miRNAs in women without breast cancer may be useful for predicting breast cancer risk and/or as an adjunct for breast cancer early detection. The miRNAs identified herein may be involved in breast carcinogenic pathways because they were first identified in the breast tissues of healthy women.

## INTRODUCTION

Breast cancer is the most common cancer among women in the US, except for non-melanotic skin cancer, and is the second leading cause of cancer-related mortality in women [1]. Knowing which women will develop breast cancer remains elusive, and currently the most effective way of addressing breast cancer morbidity and mortality is through early detection and mammography [2, 3]. Statistical models based on breast cancer risk factors have been developed to assess life-time breast cancer risk in healthy women, guiding clinical decisions for early breast cancer detection (e.g., mammography and magnetic resonance imaging) and chemoprevention [4–10]. A widely used risk assessment method is the Breast Cancer Risk Assessment Tool, typically referred to as the “Gail model” [4], and is the only model that has been repeatedly validated in large population-based studies [5, 11–13]. The Gail model incorporates age, history of breast biopsies, family history of breast cancer, and reproductive histories. However, the predictivity of the Gail model is limited, as is its application to tailoring early detection for the general population of women. Thus, improved risk assessment and/or early detection methods are needed, because many aggressive breast cancers escape detection by mammography for some women, while at the same time mammography can lead to overdiagnosis for other women [14, 15].

One approach to improve life-time breast cancer risk prediction and/or the early detection of breast cancer is to utilize molecular signatures from normal tissue, before women develop clinical abnormalities [16–18]. For example, one study showed that epigenetic markers (DNA methylation) may improve the accuracy of the Gail model [19]. Recently, miRNAs have emerged as potential biomarkers for early detection of cancer [20–24]. miRNAs are short non-coding RNAs that are abundantly present in human cells, and negatively regulate gene and miRNA expression changes in breast cancer [25–27]. In normal cells, miRNAs affect mammary gland development and other functions [28]. In breast cancer, miRNA expression is associated with diagnosis and prognosis [29–31].

In this report, we hypothesized that the identification of miRNAs in healthy women associated with breast cancer risk can be one way of developing models for breast cancer risk assessment, and/or be used as an adjunct for enhancing early detection. To address this hypothesis, two independent cross-sectional studies of women undergoing reduction mammoplasty (RM) who had no prior history of breast cancer were used to build and evaluate a multi-miRNA model for breast cancer risk (i.e., Gail risk) prediction. We subsequently analyzed the National Institute of Environmental Health Science's Sister Study cohort (a publically available data set) [21], using a nested case-control design, to assess the utility of the multi-miRNA model using serum samples to directly assess breast cancer risk. This latter study includes women without breast cancer at the time of blood draw and were either diagnosed with breast cancer within 18 months (cases), or remained without breast cancer (controls).

## RESULTS

### Participants' characteristics

The characteristics of the participants are shown in Table 1. There were 90 and 71 subjects for the discovery and independent replication studies, respectively. The median age for the discovery subjects was 45 years (range: 35-76 years) and for the replication subjects it was 46 years (range: 35-66 years). The majority of subjects were Caucasians (71.1% and 78.9%, for the discovery and replication studies, respectively), and most were classified as low risk women by the Gail model; 83.3% and 67.6%, for the discovery and replication studies, respectively). Race and Gail risk, but not other characteristics, differed significantly between the two studies (*P* = 0.0005 and *P* = 0.032, respectively).

**Table 1:** Characteristics of RM studies participants ≥ 35 y.o

Gail Characteristics	Discovery study subjects(*n* = 90) [32]		Replication study subjects(*n* = 71) [34]	
	No.	%	No.	%
Race				
White	64	71.1	56	78.9
Black	25	27.8	5	7.0
Hispanic	1	1.1	8	11.3
Other	0	1.1	2	2.8
Age, years				
< 50	62	68.9	44	62.0
≥ 50	28	31.1	27	38.0
Median	45		46	
Range	35-76		35-66	
Age at menarche, years				
< 12	15	16.7	17	24.0
12-13	43	47.7	32	45.0
≥ 14	16	17.8	20	28.2
Unknown	16	17.8	2	2.8
Age at first live birth, years				
Nulliparous	22	24.4	13	18.3
< 20	9	10.0	16	22.6
20-24	12	13.3	17	23.9
25-29	16	17.8	10	14.1
≥ 30	11	12.2	13	18.3
Unknown	20	22.2	2	2.8
No. of 1^st^ degree relatives with breast cancer				
0	57	63.3	60	84.5
1	8	8.9	9	12.7
Unknown	25	27.8	2	2.8
No. of biopsies				
0	40	44.4	65	91.6
1	6	6.7	4	5.6
≥ 2	2	2.2	2	2.8
Unknown	42	46.7	0	0
Breast Cancer riskǂ				
Low	75	83.3	48	67.6
High	15	16.7	23	32.4

ǂ High breast cancer risk was defined as woman who has at least 10% increased risk of breast cancer relative to women at average risk of the same ethnicity and similar age, estimated by the Gail model.

### miRNAs in the RM discovery and independent replication studies

**Figure 1:**
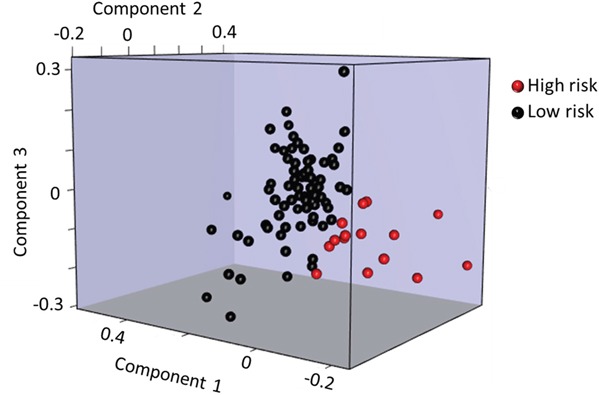
Graphical 3D representations of the women using sPLS-DA components Plot of the first 3 components of the women showing a good separation between the women with high (red) and low (black) risk of developing breast cancer as calculated by Gail model.

Five individual miRNAs (of the 168 miRNAs expressed above background) were differentially expressed in high vs. low risk women in the discovery study (*P* < 0.05), but none of these remained statistically significant after adjustment for multiple comparisons (Supplementary Table 1). To assess the applicability of miRNA-based model to predict breast cancer risk, we built a 41-miRNA model to distinguish between women with high vs. low risk of breast cancer (based upon the Gail risk model) in the discovery study (Supplementary Figures 1 and 2) using a projection-based multivariate classification technique for high dimensional data called sPLS-DA. The miRNAs in the model along with their weights are listed in Supplementary Table 2. Figure 1 shows the sPLS-DA components separating the two groups of women. The model had 83.3% predictive accuracy, 84% specificity, 80% sensitivity with 95.4% NPV and 50% PPV, in the discovery study. In an independent replication study, the same model achieved accuracy of 63.4% with specificity of 77.1% and sensitivity of 34.8%. The model had a NPV of 71.1% and a PPV of 42.1% for this data set (Table 2). The permutation test results show that our model predicteds high and low risk women with accuracies better than random chance (*P* = 0.09, Table 2 and Supplementary Information). In a sensitivity analysis, we additionally verified that classification using the 41-miRNA panel was not significantly influenced by race in the discovery RM study of women without breast cancer with 28% African American women (Supplementary Information).

**Table 2:** Classification performance of the discovery, independent replication and serum studies

Studies	Accuracy	Specificity	Sensitivity	Negative Predictive Value	Positive Predictive Value	*P*-value*
***41-miRNA model performance in breast tissue***						
Discovery	0.833	0.840	0.800	0.954	0.500	-
Replication	0.634	0.771	0.348	0.711	0.421	0.090
20-miRNA*** model performance in breast tissue and serum						
Discovery	0.811	0.813	0.800	0.953	0.461	-
Serum (GSE44281)	0.532	0.746	0.317	-	-	0.064

* *P*-value testing the performance of miRNA model based on 10,000 random permutations.

***Only 20 out of 41-miRNA were profiled and detected above background level in serum samples.

### Functional analysis of the miRNAs targets

**Figure 2:**
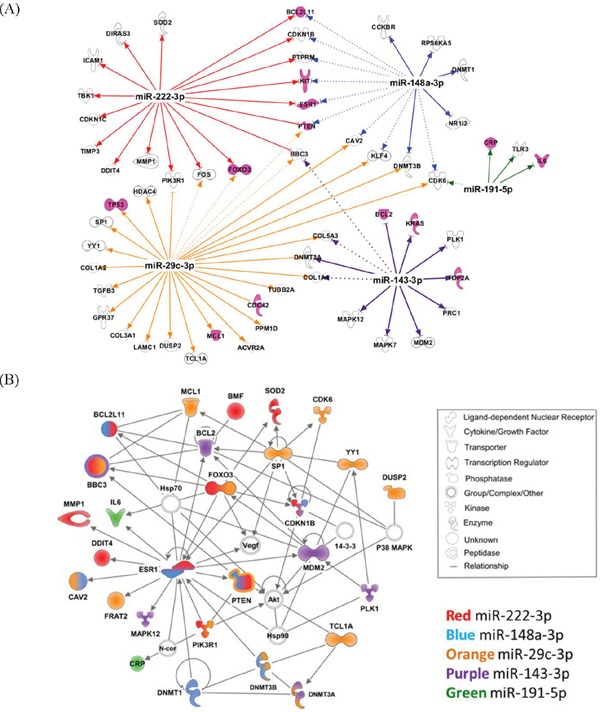
A. Five of the top 10 miRNAs have experimentally validated gene targets Gene targets involved in cancer are shown. Pink molecules are important in breast cancer pathway. The connections show experimentally validated targets (solid line) and targets predicted with high confidence (dash line). **B**. The top network of the validated gene targets is enriched in cell death and survival, cancer, liver necrosis/cell death (P =10^-50^, right-tailed Fisher's exact test). Fill colors represent molecules directly targeted by the corresponding miRNAs.

**Figure 3:**
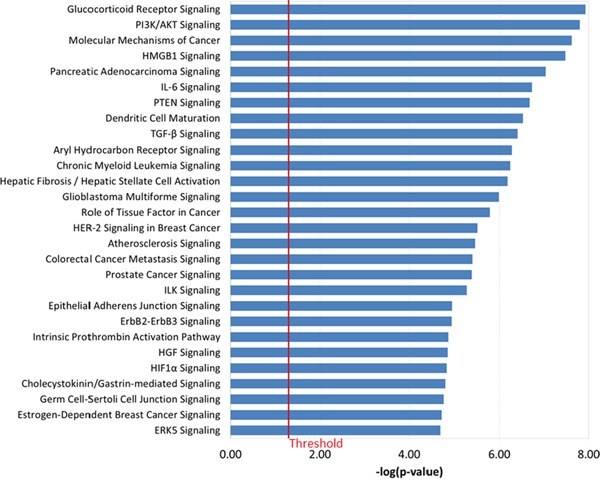
Canonical pathways that are significantly associated with the experimentally observed gene targets of the top 10 miRNA in the 41-miRNA panel using IPA Fisher's exact test was used to calculate a *P* value. Values greater than the threshold implies that the association between the miRNA gene targets and the pathway is not likely due to random chance alone.

To examine the biologic function of the miRNA panel, we performed Ingenuity Pathway Analysis (IPA) of the 41 miRNAs associated with increased Gail breast cancer risk. Given that a single miRNA can target many genes, the focus herein was on experimentally validated targets (based on the IPA knowledge base) of the top 10 miRNAs, ranked by their weights in the sPLS-DA model (Supplementary Table 2). Five out of the top 10 miRNAs had 94 experimentally validated targets. Figure 2A shows these five miRNAs and their respective gene targets that are known to be involved in cancer. The network that depicts the known direct interaction between these genes targets are shown in Figure 2B. This network is enriched in cell death and survival, cancer, and liver necrosis/cell death (*P* < 0.0001, Fisher's test). IPA canonical pathway analysis revealed significant enrichment in HER-2 signaling and estrogen-dependent breast cancer signaling, as well as other important cancer pathways such as PI3K/AKT signaling, PTEN signaling, and TGF-beta signaling (*P* < 0.0001, Figure 3 and Supplementary Table 3). Functional analysis indicated significant representation related to cellular growth and proliferation, cell death and survival, cell cycle and cancer (Supplementary Figure 3 and Supplementary Table 4).

For the other five miRNAs that had no experimentally validated targets, they had 98 predicted targets that are involved in breast cancer pathway (Supplementary Figure 4).

### miRNAs in sister study cohort

Shifting from breast tissue to serum analysis for the prediction of actual breast cancer in the Sister Study cohort using a nested case-control design (*n* = 205 cases and 205 controls), 34 of the 41 breast miRNAs identified in the discovery study were profiled in the serum of the Sister Study cohort. There were 20 of 34 miRNAs detected above background level in more than 50 women; these 20 miRNAs were then used to build a new classification model in the discovery RM set, rather than the 41 miRNAs. This model had 81.1% accuracy, 81.3% specificity, and 80% sensitivity. The 20-miRNA model was then locked and applied to classify women in the breast cancer cohort subjects. The 20-miRNA model correctly identified 74.6% of women who remained cancer-free and 31.7% of women who were diagnosed with breast cancer (Table 2). The predictive accuracy was 53.2%. It was obtained by applying breast tissue results to serum, using a different miRNA platform, and studying women with a different body habitus (the RM subjects have a higher BMI than the general population). However, the 20-miRNA model achieved better performance than random permutation (*P* = 0.06). To be applicable in the clinical setting, we derived continuous risk scores based on the sPLS-DA model prediction. Women in the highest quartile of this score had a 53% increased risk for breast cancer (odds ratio [OR] = 1.53; *P* = 0.20), albeit based on small numbers of subjects and a statistically non-significant result.

## DISCUSSION

The use of molecular markers for cancer risk prediction is rapidly increasing [23, 32–35]. This study is the first to report on the accuracy of a miRNA model developed in breast tissues of women without breast cancer to predict breast cancer risk, serving as proof-of-principle that miRNAs profiled in histologically normal breast tissue may be used to predict the risk of developing breast cancer before major carcinogenic changes and/or as a way to enhance the early detection of breast cancer. We observed that this miRNA model developed using normal breast tissue may have some predictive power to differentiate women with high and low breast cancer risk and that a subset of these miRNAs detectable in the serum could identify women who were then diagnosed with breast cancer within 18 months. The results for women without breast cancer and their breast tissues indicate that miRNAs might be useful for assessing life-time breast cancer risk (as does the Gail risk model). The analysis of serum in the case-control study of breast cancer nested within the Sister Study cohort validates the results herein as a breast cancer risk predictor, but also possibly as a marker for early detection because of the short time to breast cancer diagnosis. While there was some loss in performance as the miRNA markers identified in breast tissues were applied to serum, this would be expected as serum analysis could reflect miRNA expression from many tissues, resulting in a loss of signal. The use of serum markers as surrogates for the target organ has been previously reported [22, 36–38]. Pathway analysis of the mRNA targets of the top miRNAs identified in the model suggested enrichment for HER-2 and estrogen-dependent breast cancer signaling, and other cancer-related pathways. Our study using tissues from women with no history of breast cancer provides a unique resource for risk assessment and early detection prior to abnormalities that are clinically detectable.

miR-222-3p, one of the top-ranked miRNAs detected in the model, has been shown to target the estrogen receptor 1 gene (ESR1) and was reported to be dramatically higher in ESR1 negative breast cancer cells, inhibiting ERα expression [39]. miR-222-3p can also trigger malignant transformation by altering the expression levels of genes involved in cell death and survival, such as CAV2, PTEN, FOXO3, CDK6 and promote aggressive ER-negative breast tumors by increasing proliferation and migratory activity of breast cancer cells [40, 41]. The miR-29c-3p, another top-ranked miRNA identified in the model, has been shown to up-regulate p53 and induce apoptosis in breast cancer cell lines [42]. For the other top-ranked miRNAs, five have no validated targets but have many predicted targets related to breast cancer such as AKT, AKT1, CCND1, EGFR1, ERBB2, SRC, PTEN which have been used as breast cancer biomarkers for prognosis, diagnosis, drug efficacy, and disease progression [43–45]. Thus, these miRNAs may have clinical potential as novel breast cancer biomarkers.

Three prior cohort studies have investigated miRNA expression in prospectively collected blood samples for breast cancer risk prediction, including the Sister Study Cohort used herein [21, 46, 47]. None were based on miRNA expression in the breast, none had independent replication and the miRNAs for each study do not overlap with each other. There were other substantial differences between the study reported herein and these other studies. For example, while the Hormones and Diet in the Etiology of Breast Cancer Cohort reported 20 differentially expressed miRNAs among 133 postmenopausal breast cancer cases and 133 controls, the miRNAs were assessed in leukocytes [46]. Leukocyte miRNA expression would only affect expression for that cell type, while serum miRNA levels likely reflects a contribution of multiple organs. Similarly, the Breast Cancer Family Registry, using high risk families, reported that five microRNAs were differentially expressed in blood cells, among 20 breast cancer cases and 20 controls, but none were validated in an additional 28 case/control pair from the same study [47]. Lastly, the Sister Study Cohort utilized serum drawn only a short time before diagnosis (within 18 months and a mean of 10 months), so these results reflect an assessment for early detection rather than long term risk prediction [21]. Each study also used a different laboratory assay for miRNA detection. The miRNAs identified herein using normal tissues were not observed in the Registry Study, but four overlapped with the Sister Study Cohort results (two of them were the highest expressing miRNAs in the Sister Study, i.e., miR-181a-5p and miR-222-3p) and 3 miRNAs overlapped with the Hormones and Diet Study (i.e., miR-1991-5p, miR-145-5p, and miR-199b-5p). Whether the disparate results are due to differences in study design, blood component, or assay methodology is unclear. Currently there is no consistency in the scientific literature for which miRNAs might be predictive of breast cancer risk, however no other study has used independent validation and assessed the accuracy of a miRNA-based model.

The performance of the model applied to two independent studies is very modest albeit significantly better than random chance at *P*-value < 0.10. This most likely due to the small number of women in the discovery study (and limited number of high breast cancer risk women without breast cancer) that were used to train the model. Moreover, transition from breast tissue to blood where some miRNAs may be expressed differently, the detection method differences, the broader range of ages and BMI, and the time from blood collection to diagnosis all could influence the results.

Findings from this study should be considered in light of some limitations. The first relates to the use of the Gail risk model, which has limited accuracy, despite the fact that it is a widely used model. Other models could have been used, such as Tyrer-Cuzick [9] and BRCAPRO [48], but these have not been validated in large population studies. Another limitation is the use of reduction mammoplasty patients for discovery and replication, where the women are mostly overweight or obese, thus limiting the generalizability of results to normal weight women. However, finding concordance with the Sister Study directly minimizes concerns about the generalizability of these findings. Separately, this study was limited to the publically available data from the Sister Study, limiting our ability to directly compare our miRNA model with Gail model and to explore additional covariates and confounding (e.g., BMI).

This study approaches the discovery of breast cancer risk miRNA models by first studying breast tissues of women without breast cancer providing comprehensive molecular measurement in target tissue. The results indicate modest concordance of miRNA expression between breast tissues and serum of women who later develop breast cancer, but given the reasons discussed above while applying breast modeling results to serum in different studies, the results indicate that the novel approach used herein has the utility to provide corroborative evidence for the ultimate development of a miRNA model for predicting breast cancer risk and/or early detection. Future prospective cohort studies with large sample size and long follow-up data are needed to confirm the promise of miRNAs in breast cancer risk prediction and early detection.

## MATERIALS AND METHODS

### Study population and biospecimen collection

#### Reduction mammoplasty (RM) discovery study

Healthy women with no prior history of breast cancer who underwent RM were studied, as described previously [49, 50]. Briefly, subjects aged 35 and older were recruited at Georgetown University Medical Center (Washington, DC), the University of Maryland (College Park, MD), the Washington Hospital Center (Washington, DC) and the Center for Plastic Surgery (Buffalo, NY) from 1997 to 2009. Recorded data by personal interview included demographics, lifestyle, reproductive history, family medical history, diet, and other exposures. Upon pathological review, subjects with gross pathology, epithelial hyperplasia, or focal microcalcifications were excluded. Tissues were dissected to remove adipose tissue, fixed in formalin, and embedded in paraffin.

#### RM independent replication study

Women ages ≥35 who underwent RM at Baystate Medical Center (Springfield, MA) between 2007 and 2009 were studied, as previously described [51]. Participants were interviewed by phone following surgery and data available from the questionnaires were similar to those in the discovery study. Breast tissues were collected similarly, except that the breast epithelial tissue was not dissected from fat before storage. Participants with benign biopsy results also were excluded.

#### Cohort study of breast cancer - Sister Study

A cohort study for breast cancer where there are publically-available serum-based miRNA profiles were then analyzed in relation to miRNA model identified from the above RM discovery study, using a nested case-control study design. The National Institute of Environmental and Health Sciences (NIEHS) Sister Study (NCBI Gene Expression Omnibus, accession number GSE44281) [21] includes 50,844 women from US or Puerto Rico who never had breast cancer but had a sister diagnosed with breast cancer. Baseline serum samples of 205 women without breast cancer who were subsequently diagnosed with breast cancer within 18 months following blood collection (mean = 10 months) were matched with 205 women who remained cancer free. The matching criteria were no prior history of cancer except non-melanoma skin cancer, race, age, date of blood draw and available blood sample. For this cohort, miRNA expression levels were determined using GeneChip miRNA 2.0 arrays (Affymetrix Inc., Santa Clara, CA, USA) as described by the original authors [21].

All participants in the three studies provided informed consent and studies were approved by the Institutional Review Boards of all participating institutions.

### RNA extraction and miRNA profiling in breast tissues

Total RNA was extracted from the two RM studies using formalin-fixed paraffin embedded (FFPE) tissues according to the manufacturer's instructions (FFPE RNA purification kit, Norgen, Canada). miRNA expression was quantified using the nCounter digital detection miRNA expression assay (NanoString^®^ Technologies, Seattle WA), with 10 % duplicates for quality control purposes. Coefficients of variation (CV) were calculated to assess assay reliability (3.76% and 4.62% in RM discovery and replication studies, respectively).

### RM subjects breast cancer risk assessment

For the RM studies, each woman's Gail score was calculated [10]. We defined “high risk” women as those with a 10% or greater increased risk relative to women of the same race and age who is at average risk (e.g. > 1.1% risk if the average 5-year risk is 1%). We refer to low risk as average risk in the population for a given age and racial/ethnic demographic. Five year risks were calculated using the latest update (May 2011) of source code for the breast cancer risk calculation engine [52]. We verified the risks calculated using the source code with risks calculated using the online tools for 30 random women. All risk estimates showed complete agreement.

### Data analysis

**Figure 4:**
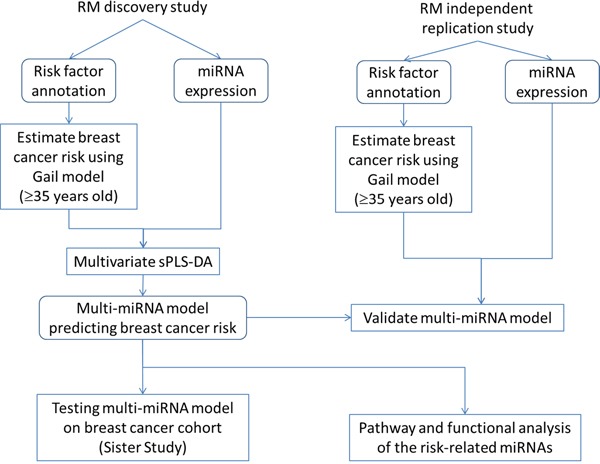
Workflow of the data analysis performed in this study Abbreviation: sPLS-DA, sparse partial least square discriminant analysis.

Figure 4 shows an overview of the data analysis performed in this study. Raw expressions were normalized using the top quartile mean normalization method [53], which has been shown to be more sensitive and accurate than using invariant miRNA [54] or quantile normalization [55, 56] (Supplementary Figure 5). 800 miRNAs were measured using the NanoString nCounter platform [57]. We filtered out miRNAs whose expressions were below NanoString's internal negative control in at least 50% of the samples, leaving 168 for downstream analysis. Principal Component Analysis (PCA) was used to detect the presence of potential confounding factors including technical artifacts and batch effects. In our miRNA dataset, no confounding was found (Supplementary Figure 6).

To confirm that patient characteristics in the RM studies were similar, chi-squared tests were used to compare the categorical characteristics between participants in the two studies. Two-sided Wilcoxon rank sum tests were used to compare expression of each miRNA individually between high and low risk women. *P*-values were corrected for multiple testing using the Benjamini-Hochberg False Discovery Rate (FDR) algorithm. [58] An FDR value less than 0.10 was considered statistically significant.

In order to build a multi-miRNA model associated with breast cancer risk in the discovery study, we used a projection-based multivariate sparse partial least squares discriminant analysis (sPLS-DA [59]) approach to classify women into low and high breast cancer risk groups. sPLS-DA was designed to classify high dimensional data that also performed variable selection [59]. Optimal parameters tuning was performed based on 10-fold cross validation (CV) to avoid overfitting [60].

In order to replicate the findings of the RM discovery study, the miRNA model which was developed in the discovery study was used to predict the breast cancer risks of women in the replication study. The accuracy, sensitivity, specificity, positive predictive value (PPV), and negative predictive value (NPV) of the miRNA model were calculated to evaluate the discriminatory performance of the miRNA panel in classifying women with low versus high Gail risk [60]. To assess whether our model is better than random chance, we applied a permutation test by reshuffling the labels, applying the same model 10,000 times and calculating the p-value. The null distribution of the accuracies given by the permutation is the accuracy of the model when it is a random signature. Small p-value indicates that the accuracy of the model is significantly better than random chance.

To assess whether the miRNA panel can prospectively predict breast cancer risk using serum in the Sisters Study, miRNAs by Affymetrix^®^ used for the Sister Study and NanoString for the RM studies were matched based on their sequences to identify available miRNAs for further analysis. Since only 20 out of 41 miRNAs were detected above background by the Affymetrix method in serum, we used these 20 miRNA to build a sPLS-DA model in the discovery study. This model was then applied to classify women into breast cancer and cancer-free categories in the Sister Study cohort. Finally, the same permutation test as described above was applied to assess whether the performance of our model was better than what chance alone could produce. Continuous risk scores were derived by taking the difference of the two outcomes variables predicted by sPLS-DA. Logistic regression was used to estimate breast cancer odds ratios (OR) and 95% confidence intervals (CI) for comparisons of the second through fourth quartiles of the risk score relative to the first. All analyses described in this section were performed in the R statistical environment v3.1.1. Further details are available in the Supplementary Information.

### Functional and pathway analysis

Functional analysis was performed using QIAGEN's Ingenuity Pathways Analysis (IPA^®^ QIAGEN Redwood City, www.ingenuity.com). miRNA targets were identified using TarBase [61], miRecords [62], TargetScan [63] and Ingenuity^®^ knowledge base. Methodology and approaches are described in detail in Supplementary Information.

## SUPPLEMENTARY INFORMATION FIGURES AND TABLES




