# Supplementary material for: Discovery and replication of microRNAs for breast cancer risk using genome-wide profiling
Source: Oncotarget. 2016 Nov 9;7(52):86457–68. doi: 10.18632/oncotarget.13241 (PMC5349926; doi:10.18632/oncotarget.13241)
Supplement: Supplementary file 1 [file oncotarget-07-86457-s001.pdf]

Discovery and replication of microRNAs for breast cancer risk using genome-wide profiling

SUPPLEMENTARY INFORMATION

NanoString® data pre-processing and analysis

One important step of preprocessing miRNA profiling data is to apply optimal normalization method. The purpose of data normalization is to minimize systematic bias due to technical variation. Normalization is very critical for getting the true biological signal, however, normalization method for miRNA data continues to be very challenging due to the lack of invariant stable miRNA [1]. At present, there is little consensus on normalization methods for pre-processing of miRNA expression [1]. In our study, we used two-step normalization where technical normalization was performed based on internal positive controls followed by global mean normalization [2] as recommended by NanoString. A global mean normalization strategy has been shown to be the most sensitive and accurate approach for miRNA normalization compared to normalization using multiple most stably miRNAs [3] or quantile normalization [4]. Additionally, we compared the two-step normalization with the commonly used quantile normalization [5] and found the two step normalization to be more precise since they have lower variance (CV) across duplicates (Supplementary Figure 1).

Specifically, the raw NanoString data were subjected to both technical normalization and global mean normalization. First, technical normalization was performed to minimize the impact of lane-to-lane variations that could arise during processing. Raw data was normalized by making the geometric mean of the positive internal control (synthetic miRNA sequences) counts to be the same across all samples. Geometric mean was used since it is more robust to outliers [6]. After the data was normalized for processing variability, it was necessary to further normalize for RNA content. Ideally, assays that were going to be compared should be performed with the same total amount of RNA with the same total amount of RNA. However, the RNA content in an assay was frequently affected by small inaccuracies in quantification or pipetting. Since invariant miRNAs were not well defined, we normalized the counts to the top 50 miRNAs with the highest mean expression across all samples (excluding problematic miRNAs, the one with +++). Specifically, we normalized the counts so that the geometric means of the top 50 miRNAs were the same across all samples.

We filtered out miRNAs that do not have signals in most of the samples (e.g., counts [before global normalization] less than background detection threshold in more than 50% of the total samples). A 168 miRNA subset passed the filter. Background thresholds were calculated for each sample as the mean of internal negative controls (unique probes with non-human target sequences) + 2 standard deviation (SD). Additionally, after global normalization, samples with very low overall counts (those with normalization factor > 10) were also filtered out. These samples have substantially less miRNA content (less efficient ligation and counting) than all the others that maybe due to sample degradation or contaminants in the RNA.

Comparison with quantile normalization

To make sure we have selected the appropriate normalization methods, we normalized our data using the method described above and compared it with data normalized using quantile normalization [5] which was commonly used to normalize gene expression data. Unsupervised hierarchical clustering was performed to compare the two normalization methods on samples that were assayed in duplicates. The coefficients of variations (CVs) of the normalized expression are shown in Supplementary Figure 1. As shown in Supplementary Figure 1, after the two step normalization, duplicates have lower variance than quantile or no normalization. This suggests that normalization is necessary and that our normalization strategy performs better than quantile normalization. Our decision to not use quantile normalization is also supported by Prokopec et al [4] which found quantile normalization removing correlation with quantitative real-time PCR (qPCR) results.

Quality control

We used principal component analysis (PCA) as a QC tool to detect the presence of batch effects and other technical artifacts that can bias our analysis. The first thing we would like to make sure is that miRNAs do not cluster together because of normalization factor. Supplementary Figure 2 shows the first two components. We can see that there is no clear separation between high and low normalization factors which indicate no evidence of confounding by normalization factor. We have also check

for batch effects due to cartridge (data not shown) and did not find any obvious patterns.

Checking for confounders

We used PCA to identify variables that represent potential confounders. First, PCA plots of the first and second component were used as exploratory step to identify potential confounders for each of our risk factors (e.g. race, dichotomized age). We do not see any clear separation of our samples which indicate that none of the risk factors in our data is a confounding factor (data not shown except for Race which was plotted in Supplementary Figure 2). Supplementary Figure 2 displays one example of the PCA plot with the color indicating race. This plot shows that race is not a confounding variable.

Univariate analysis

Chi-squared tests were used to compare the categorical characteristics factors between participants in the two RM studies. Categorical characteristics for “unknown” status were excluded in the Chi-squared tests. In the case where the expected counts were small, the sampling distribution of the chi-squared statistics does not follow the chi-squared distribution. In such cases, we used Monte Carlo to simulate distribution of the chi-squared statistics [9]. Wilcoxon rank sum tests were used to compare expression of each miRNA individually between high and low risk women. P-values were adjusted for multiple comparisons using the Benjamini-Hochberg False Discovery Rate (FDR) algorithm [10].

miRNA model identification (Multivariate analysis)

Multivariate analysis, Sparse Partial Least Square Discriminant Analysis (sPLS-DA) was done using mixOmics (4.0-2) package in R [11, 12]. sPLS-DA, an extension of PLS is a class of technique where two matrices, X and Y are modeled by latent variables coupled with variable selection and classification a one-step procedure. sPLS-DA is especially useful when analyzing correlated highly dimensional data or when there are more variables than observation. The sparsity extension of PLS-DA is motivated by the need to separate the biological signal from non-useful noisy data. Dimension reduction was achieved by singular value decomposition (SVD) computation and by penalizing the sparse SVD. In this case, miRNAs selection was integrated with modeling as a one-step procedure and the aim is to model a reciprocal relationship between miRNA expression and Gail risk [13]. First, latent components were constructed using sPLS regression by converting categorical response to dummy coding $\{0, 1\}$. Hence the sPLS model is given by:

$$\boldsymbol{Y} = \boldsymbol{TQ}^T + \boldsymbol{F}$$

$$\boldsymbol{X} = \boldsymbol{TP}^T + \boldsymbol{E}$$

$$\boldsymbol{T} = \boldsymbol{XW}$$

Where $\boldsymbol{Y}$ and $\boldsymbol{X}$ represent the response and the predictor matrices, respectively. $\boldsymbol{Q}$ and $\boldsymbol{P}$ are coefficients (loadings) and $\boldsymbol{E}$ and $\boldsymbol{F}$ are errors. $\boldsymbol{T}$ is the latent components underlying both $\boldsymbol{Y}$ and $\boldsymbol{X}$. $\boldsymbol{W}$ are the K direction vectors which are solve along with the variable selection.

Variable selection was incorporated by solving the following optimization problem:

$$\min_{w,c} -k\boldsymbol{w}^{\boldsymbol{T}}\boldsymbol{M}\boldsymbol{w} + (1-k)(\boldsymbol{c}-\boldsymbol{w})^{\boldsymbol{T}}\boldsymbol{M}(\boldsymbol{c}-\boldsymbol{w}) + \lambda_1\|\boldsymbol{c}\|_1 + \lambda_2\|\boldsymbol{c}\|_2$$

Where $\boldsymbol{M} = \boldsymbol{X}^T\boldsymbol{Y}\boldsymbol{Y}^T\boldsymbol{X}$. This objective function can be interpreted as maximizing the covariance between $\boldsymbol{X}$ and $\boldsymbol{Y}$ and incorporate variable selection by imposing L1 penalty onto a surrogate of direction vector $\boldsymbol{c}$ instead of the original direction vector **w**, while keeping $\boldsymbol{w}$ and $\boldsymbol{c}$ close to each other. The last step is applying linear discriminant analysis (LDA). Let $\hat{\beta}^{LC}$ be the coefficient estimates of the latent components. Then, the original predictors can be obtained as $\hat{\beta} = \boldsymbol{W}\hat{\beta}^{LC}$ because $\boldsymbol{T}\hat{\beta}^{LC} = \boldsymbol{X}\boldsymbol{W}\hat{\beta}^{LC} = \boldsymbol{X}\hat{\beta}$. The class prediction were then determined based on largest predicted value. More detailed formulation of the sPLS-DA model can be found in Chung and Keles [14].

Parameters tuning for sPLS-DA were done by evaluating the performance classification during 50 iterations of 10-fold cross validation. During this procedure, the RM discovery study was randomly split into 10 equal subsets. Model estimation was conducted on nine tenths of the study, and tested on the remaining tenth of the study that were held out. This was repeated until each group of a tenth of the study played the role of test samples once and the whole process was repeated 50 times. The cross validation results showed that the best model (the non-parsimonious, most stable model with an average error rate of 0.099) was one with 41 miRNAs and 3 components. Supplementary Figure 3 shows plot of the classification error during cross validation. 41-miRNA model was identified based on the miRNAs which make up the first component as this component corresponds to 91% variation explained (Supplementary Figure 4).

RM discovery and replication studies

Once the 41-miRNAs were identified, we used the same miRNAs to build sPLS-DA model in the RM

discovery and applied it to the replication studies. Because of potential batch effects, we standardized the miRNA measurements within each study. Expressions were centered by subtracting the means and dividing the (centered) expressions by their standard deviations. Accuracy, sensitivity, and specificity of the prediction results were calculated. We referred to agreement to Gail risk as accuracy. To assess whether the classification error rates differ significantly from what chance alone could produce, a permutation test was applied. In a permutation test, outcome labels (high vs. low risk) were permuted and assigned randomly to each woman. The same model developed in the discovery study was then applied to these permuted samples and classification accuracy was calculated. This procedure was repeated 10,000 times to get the distribution of permuted accuracy as the null distribution. The P-value of the accuracy was calculated by summing the number of permuted accuracies > the accuracy obtained on the full study set divided by 10,000. Results are shown in Table 2.

Sensitivity analysis

Because race distribution in the two studies was different we performed a sensitivity analysis to test whether classification based on the 41-miRNA panel was significantly influenced by race. We performed a logistic regression with dichotomized Gail risk as the dependent variable and predicted Gail risks (using 41-miRNA panel) as independent variable, with and without race adjustment. The *p*-values for predicted Gail is 2.33×10^{-5} (odd ratio [OR] = 21.92) and 2.24×10^{-5} (OR = 21.01) with and without race adjustment, respectively. Therefore we concluded that race is likely not a major confounding factor ($P > 0.3$) in the association between the miR model and dichotomized Gail risk.

miRNA signature as non-invasive markers (Sister study)

In order to assess the capability of the identified miRNA signature in predicting real breast cancer cases, we used public dataset (GSE44281) of miRNA profiles in serum from 410 women without breast cancer (205 remained cancer-free and 205 developed breast cancers). miRNA expression profiled using Affymetrix® array were background corrected and normalized using robust multichip average (RMA) method [15]. R package 'GEOquery' [16] was used to access the NCBI Gene Expression Omnibus (GEO) public repository. To avoid confusion due to changes in miRNA naming systems according to which miRbase was referenced, we matched Affymetrix probe set names and NanoString probe names based on their sequences. 34 out of the 41 miRNAs identified in discovery study were covered in Affymetrix array. Twenty out of 34 miRNAs were detected above background level in more than 50 women. *P*-value of less than 0.06 (Wilcoxon rank-sum test) was used to identify miRNAs above background level as recommended by the manufacturer. The same filter criteria were also used in the analysis of Sister Study [17]. We built a classification model based on these 20 miRNAs in the discovery study using sPLS-DA. Because Sister Study used a different platform to measure the miRNA profiles, we standardized the miRNA expression. We then used the 20-miRNA model to identify women who remain cancer-free and those who later develop cancer. To assess whether the classification error rates differ significantly from what chance alone could produce, a permutation test was applied. In a permutation test, outcome labels (develop cancer vs. remain cancer-free) were permuted and assigned randomly to each patient. The 20-miRNA model was used to classify women into cancer and cancer-free groups in the permuted samples. This procedure was repeated 10,000 times to get the null distribution of accuracies which was then compared to accuracy obtained in the Sister Study. Results are shown in Table 2.

IPA analysis

Data were analyzed using Ingenuity Pathways Analysis (IPA; Ingenuity Systems, Redwood City, CA, www.ingenuity.com) and described below. Experimentally validated miRNA targets were identified using TarBase [18], miRecords [19] and Ingenuity® knowledge base. Predicted targets with high confidence were determined using TargetScan [20]. All miRNA targets were identified using the previously mentioned databases from within IPA.

Network generation, canonical pathway and functional analysis

A data set containing the top 10 miRNA identifiers (based on weights calculated by sPLS-DA) was uploaded into IPA. Each miRNA identifier was mapped to its corresponding miRNA object. Only 5 out of the top 10 miRNAs have experimentally validated targets. 94 experimentally validated gene targets of these 5 miRNAs were identified using TarBase [18] and miRecords [19]. Networks of these target genes were then algorithmically generated based on connectivity information contained in the IPA knowledge base. *P*-values were calculated using the right-tailed Fisher's exact test determining the probability of getting the same network by chance when randomly picking 94 molecules that can be in the networks.

The functional analysis identified the biologic functions and/or diseases that were most significantly associated with the 94 experimentally validated target

genes of the top 10 miRNA in the model. The canonical pathway analysis identified the pathways from the IPA library of canonical pathways that were most significant to the data set. The significance of the association between the 94 validated target genes and the canonical pathway or the biologic functions were measured in two ways: (1) *P*-values were calculated using Fisher's exact test determining the probability that the association between the target genes and the canonical pathway (or the biologic functions) is explained by chance alone and (2) a ratio of the number of target genes that map to the pathway to the total number of genes in the canonical pathway (or in the diseases and functions category). These are shown in the Supplementary Tables 3 and 4.

REFERENCES

1. Meyer SU, Pfaffl MW, Ulbrich SE. Normalization strategies for microRNA profiling experiments: a "normal" way to a hidden layer of complexity? *Biotechnol Lett*. 2010;32:1777-1788. doi:10.1007/s10529-010-0380-z.
2. Mestdagh P, Van Vlierberghe P, De Weer A, et al. A novel and universal method for microRNA RT-qPCR data normalization. *Genome Biol*. 2009;10:R64. doi:10.1186/gb-2009-10-6-r64.
3. D'haene B, Mestdagh P, Hellemans J, Vandesompele J. miRNA expression profiling: from reference genes to global mean normalization. *Methods Mol Biol*. 2012;822:261-272. doi:10.1007/978-1-61779-427-8_18.
4. Prokopec SD, Watson JD, Waggott DM, et al. Systematic evaluation of medium-throughput mRNA abundance platforms. *RNA*. 2013;19:51-62. doi:10.1261/rna.034710.112.
5. Bolstad BM, Irizarry R., Astrand M, Speed TP. A comparison of normalization methods for high density oligonucleotide array data based on variance and bias. *Bioinformatics*. 2003;19:185-193. doi:10.1093/bioinformatics/19.2.185.
6. Van Belle G, Fisher L. *Biostatistics: A Methodology for the Health Sciences*. John Wiley & Sons; 2004. http://books.google.com/books?id=MZhqAAAAMAAJ.
7. Matsuno RK, Costantino JP, Ziegler RG, et al. Projecting individualized absolute invasive breast cancer risk in Asian and Pacific Islander American women. *J Natl Cancer Inst*. 2011;103:951-961. doi:10.1093/jnci/djr154.
8. Breast Cancer Risk Assessment C# program. http://www.cancer.gov/bcrisktool/download-source-code.aspx.
9. Hope ACA. A Simplified Monte Carlo Significance Test Procedure. *J R Stat Soc Ser B*. 1968;30:582-598. doi:10.2307/2984263.
10. Benjamini Y, Hochberg Y. Controlling the False Discovery Rate: A Practical and Powerful Approach to Multiple Testing. *J R Stat Soc Ser B*. 1995;57:289-300. doi:10.2307/2346101.
11. Dejean S, Gonzalez I, with contributions from Pierre Monget K-ALC, Coquery J, Yao F, Liquet B. mixOmics: Omics Data Integration Project. 2013. http://cran.r-project.org/package=mixOmics.
12. R Core Team. R: A Language and Environment for Statistical Computing. 2012. http://www.r-project.org/.
13. Lê Cao K-A, Boitard S, Besse P. Sparse PLS discriminant analysis: biologically relevant feature selection and graphical displays for multiclass problems. *BMC Bioinformatics*. 2011;12:253. doi:10.1186/1471-2105-12-253.
14. Chung D, Keles S. Sparse partial least squares classification for high dimensional data. *Stat Appl Genet Mol Biol*. 2010;9:Article17. doi:10.2202/1544-6115.1492.
15. Irizarry RA, Hobbs B, Collin F, et al. Exploration, normalization, and summaries of high density oligonucleotide array probe level data. *Biostatistics*. 2003;4:249-264. http://biostatistics.oxfordjournals.org/content/4/2/249.abstract.
16. Davis S, Meltzer PS. GEOquery: a bridge between the Gene Expression Omnibus (GEO) and BioConductor. *Bioinformatics*. 2007;23:1846-1847. doi:10.1093/bioinformatics/btm254.
17. Godfrey AC, Xu Z, Weinberg CR, et al. Serum microRNA expression as an early marker for breast cancer risk in prospectively collected samples from the Sister Study cohort. *Breast Cancer Res*. 2013;15:R42. doi:10.1186/bcr3428.
18. Vergoulis T, Vlachos IS, Alexiou P, et al. TarBase 6.0: capturing the exponential growth of miRNA targets with experimental support. *Nucleic Acids Res*. 2012;40:D222-D229. doi:10.1093/nar/gkr1161.
19. Xiao F, Zuo Z, Cai G, Kang S, Gao X, Li T. miRecords: an integrated resource for microRNA-target interactions. *Nucleic Acids Res*. 2009;37:D105-D110. doi:10.1093/nar/gkn851.
20. Friedman RC, Farh KK-H, Burge CB, Bartel DP. Most mammalian mRNAs are conserved targets of microRNAs. *Genome Res*. 2009;19:92-105. doi:10.1101/gr.082701.108.

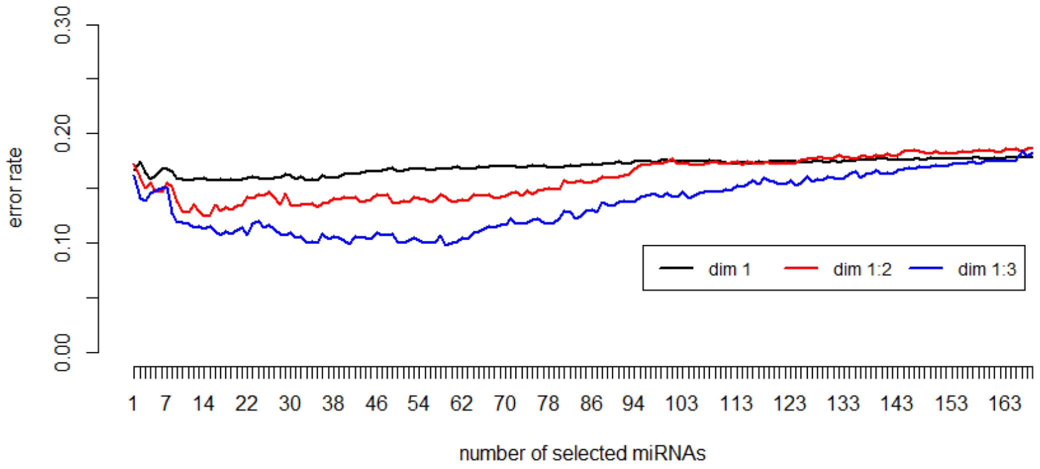

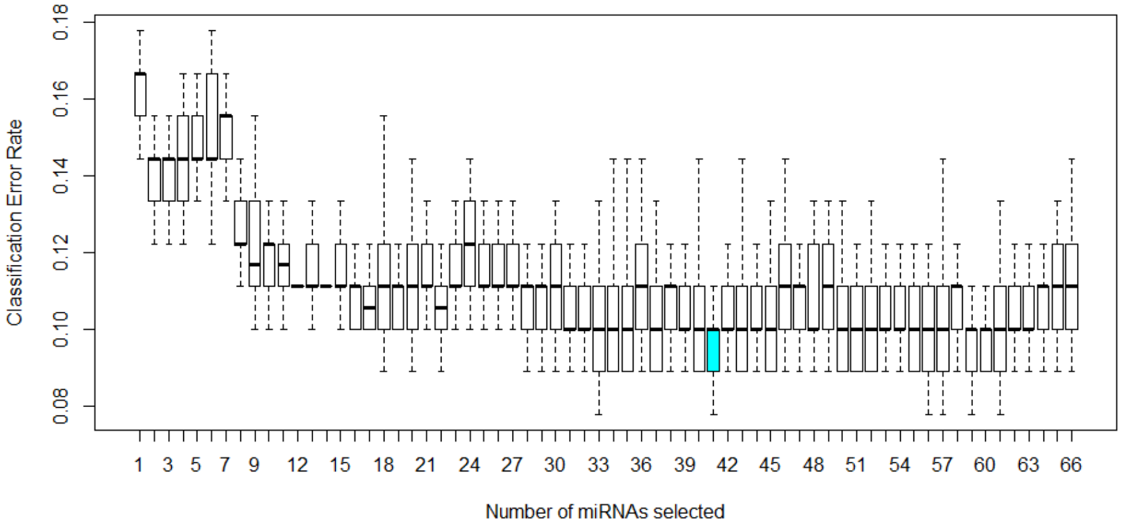

Supplementary Figure 1: Sensitivity analysis on the number of selected miRNAs and the number of dimension chosen calculated using 50 iterations of 10-fold cross validation. Model with 41 miRNAs is the simplest model with the best performance (colored in cyan).

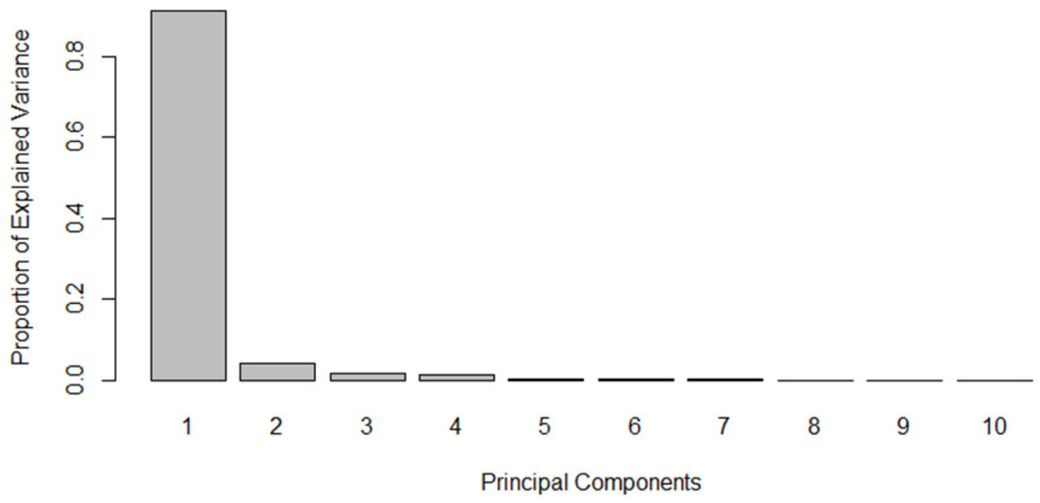

Supplementary Figure 2: Plot of cumulative proportion of explained variance for the first 10 principal components. The first component can explain up to 91% of the variation. Thus, we decided to select the 41 miRNAs which make up the first component.

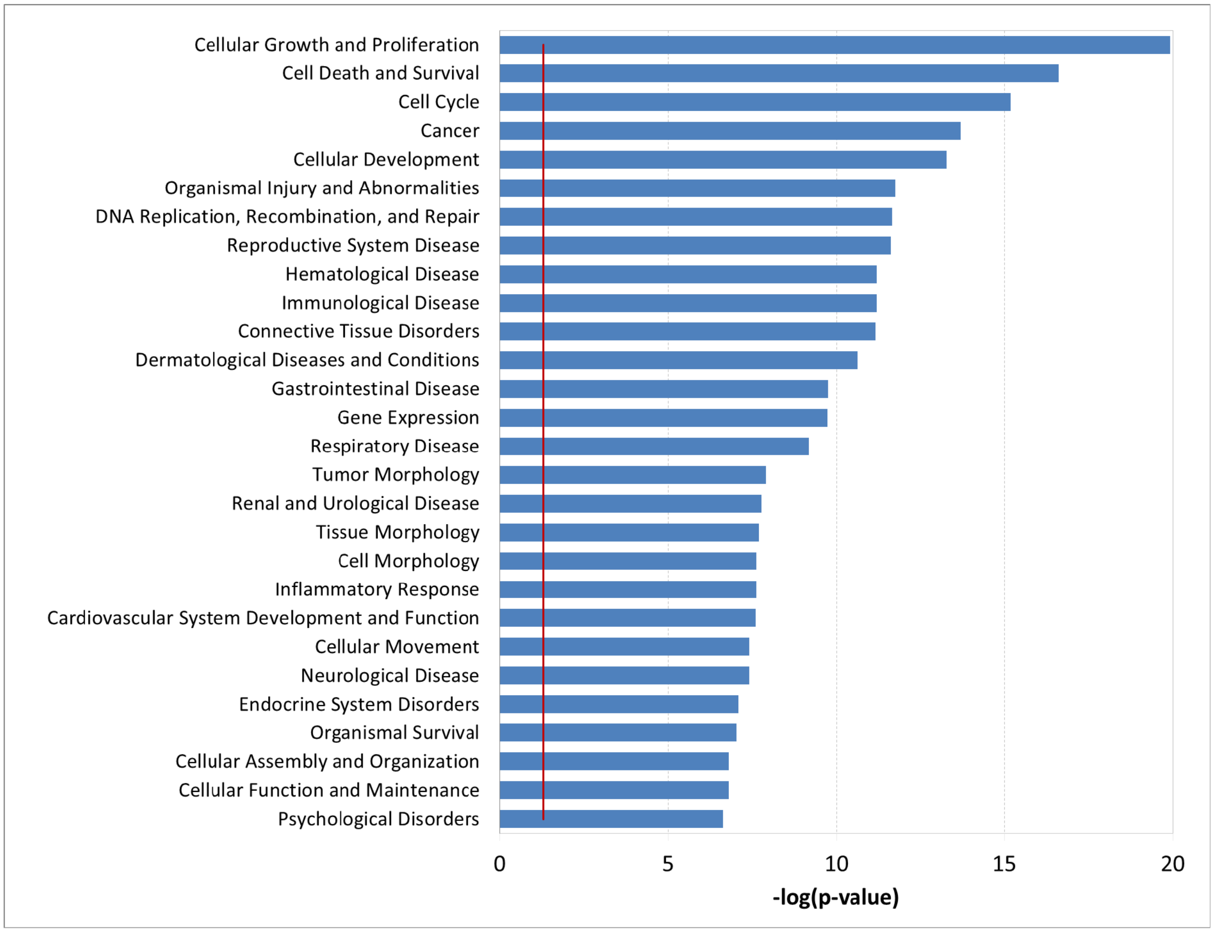

Supplementary Figure 3: IPA functional analysis associated with the experimentally validated gene targets of the top 10 miRNA in the 41-miRNA signature. Fisher's exact test was used to calculate *P*-values. Values greater than the threshold (red vertical bar) implies that the association between miRNA gene targets and the functions is not likely due to random chance alone.

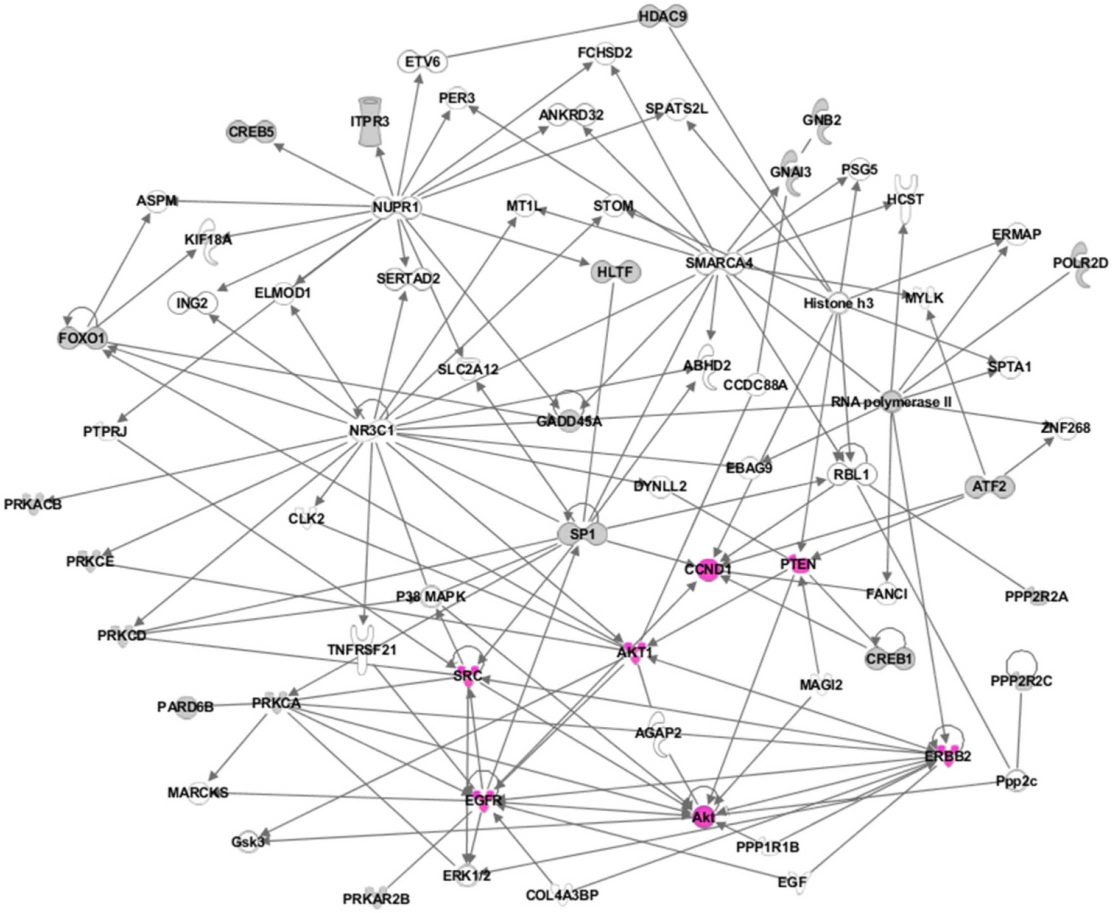

Supplementary Figure 4: Network of predicted targets (shaded molecules) of top 10 miRNAs with no experimentally validated targets that are involved in breast cancer pathway. Pink molecules are important in breast cancer pathway. Network is enriched in cell cycle, cell death and survival, and post-translational modification ($P = 10^{-50}$, right-tailed Fisher's exact test).

A.

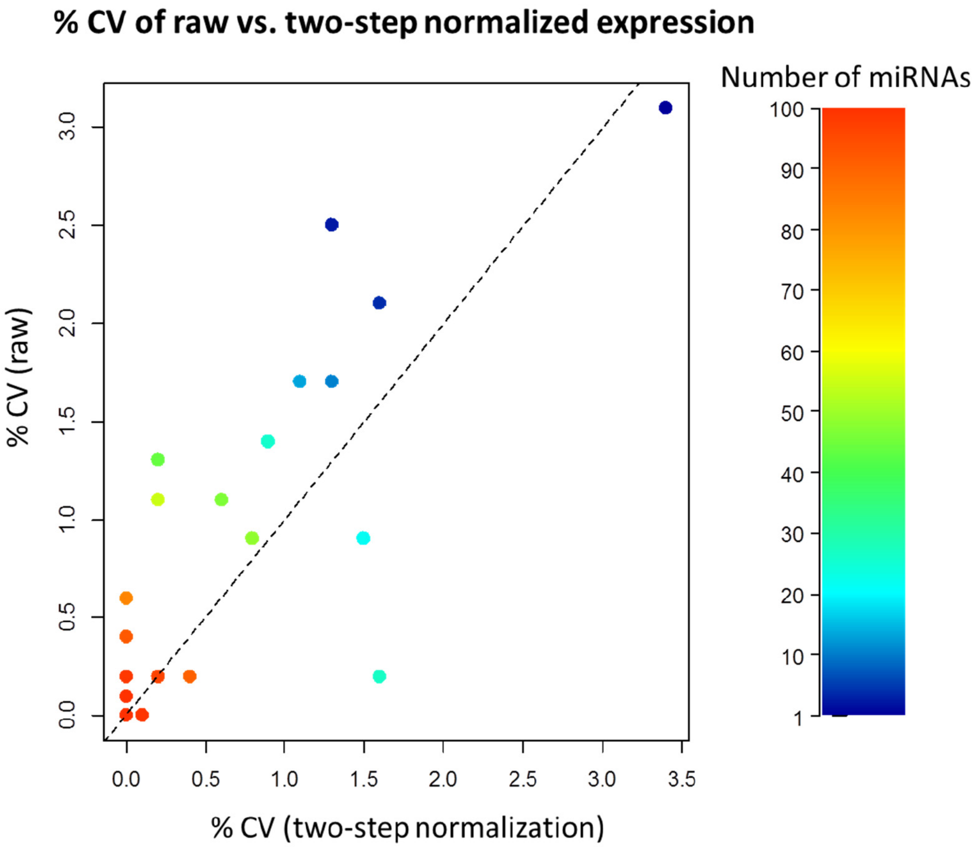

B.

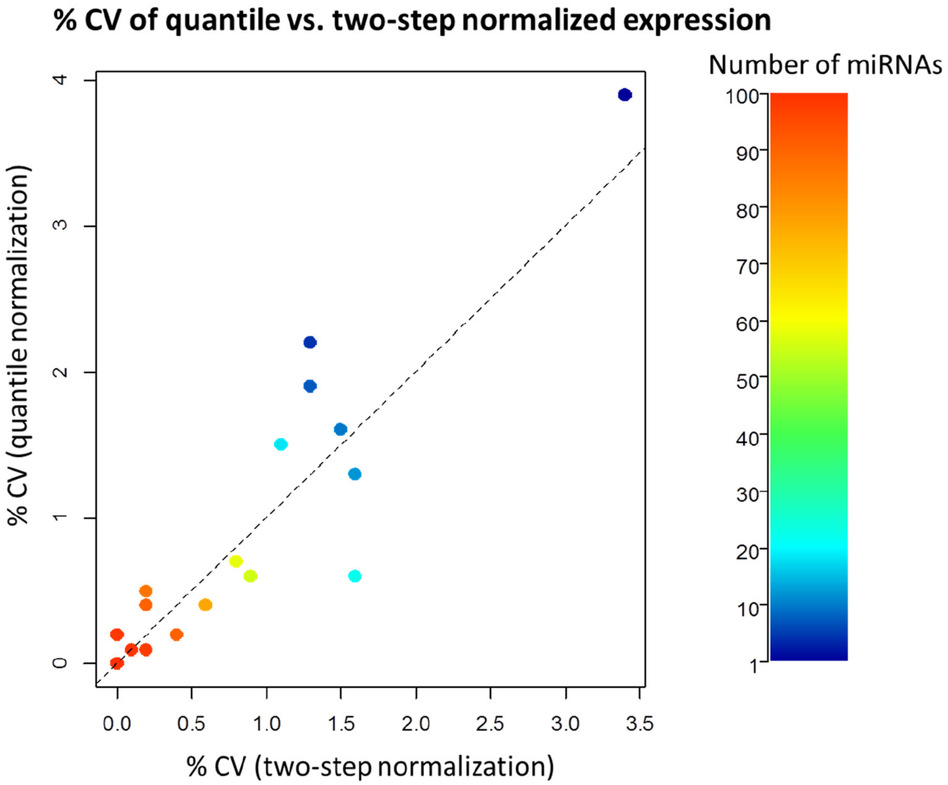

Supplementary Figure 5: Comparison of CV of miRNA expressions A. raw vs. two-step normalization and B. quantile normalization vs. two-step normalization. Each point represents miRNA(s). Colors represent number of miRNAs with the same CVs. These figures show that after two-step normalization, miRNA expressions of duplicates are more similar than after quantile or no normalization.

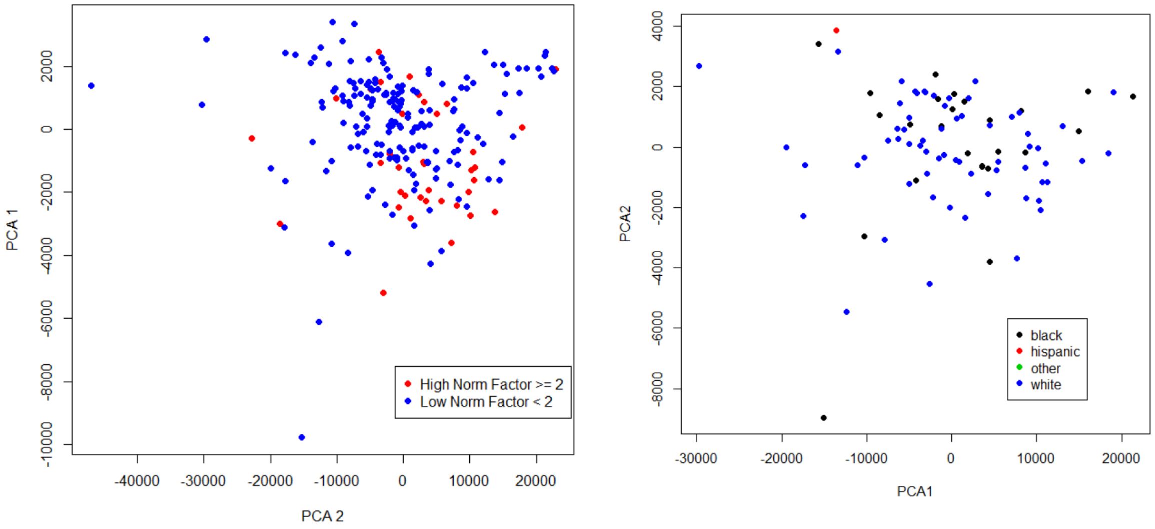

Supplementary Figure 6: PCA plot of the first two components with the samples colored according to their normalization factor (left) and race (right). This plot indicated there are no technical variations due to the different normalization factor and that race is not a confounding factor in our miRNA expression.

Supplementary Table 1: Univariate analysis of miRNA expression in low and high risk women

miRNA	Mean miRNA low-risk (log_2)	Mean miRNA high-risk (log_2)	Fold-Change	FDR	P-value
hsa-miR-148a-3p	6.39	5.96	-1.34	4.72E-01	2.81E-03
hsa-miR-29c-3p	5.42	5.16	-1.20	7.65E-01	1.67E-02
hsa-miR-143-3p	7.71	6.95	-1.68	7.65E-01	2.65E-02
hsa-miR-204-5p	4.59	4.31	-1.21	7.65E-01	4.07E-02
hsa-miR-374b-5p	4.76	5.1	1.26	7.65E-01	4.29E-02

Supplementary Table 2: List of 41-miRNA signature for predicting women with low- and high breast cancerrisk sorted by decreasing estimated loadings (weights) (Bolded miRNAs are the 20 miRNAs detectable in serum samples. Loadings shown was calculated in the discovery study for the first component in standardized unit.)

See Supplementary File S1.

Supplementary Table 3: IPA significant canonical pathways

See Supplementary File S1.

Supplementary Table 4: IPA significant biologic functions and/or diseases

See Supplementary File S1.
