# Supplementary material for: Discovery and replication of microRNAs for breast cancer risk using genome-wide profiling
Source: Oncotarget. 2016 Nov 9;7(52):86457–68. doi: 10.18632/oncotarget.13241 (PMC5349926; doi:10.18632/oncotarget.13241)
Supplement: Supplementary file 2 [file oncotarget-07-86457-s002.docx]

| Supplementary Table 2. List of 41-miRNA signature for predicting women with low- and high breast cancerrisk sorted by decreasing estimated loadings (weights) (**Bolded** miRNAs are the 20 miRNAs detectable in serum samples. Loadings shown was calculated in the discovery study for the first component in standardized unit.) | | | | |
| --- | --- | --- | --- | --- |
|  | miRNA names | Loadings | Accession | Target sequence |
| 1 | hsa-miR-148a-3p | -0.41 | MIMAT0000243 | UCAGUGCACUACAGAACUUUGU |
| 2 | hsa-miR-374b-5p | 0.40 | MIMAT0004955 | AUAUAAUACAACCUGCUAAGUG |
| 3 | hsa-miR-548ah-5p | 0.30 | MIMAT0018972 | AAAAGUGAUUGCAGUGUUUG |
| 4 | hsa-miR-548ad | 0.28 | MIMAT0018946 | GAAAACGACAAUGACUUUUGCA |
| 5 | **hsa-miR-1972** | 0.27 | MIMAT0009447 | UCAGGCCAGGCACAGUGGCUCA |
| 6 | hsa-miR-29c-3p | -0.25 | MIMAT0000681 | UAGCACCAUUUGAAAUCGGUUA |
| 7 | **hsa-miR-143-3p** | -0.24 | MIMAT0000435 | UGAGAUGAAGCACUGUAGCUC |
| 8 | hsa-miR-191-5p | -0.21 | MIMAT0000440 | CAACGGAAUCCCAAAAGCAGCUG |
| 9 | **hsa-miR-222-3p** | 0.20 | MIMAT0000279 | AGCUACAUCUGGCUACUGGGU |
| 10 | **hsa-miR-151a-3p** | 0.19 | MIMAT0000757 | CUAGACUGAAGCUCCUUGAGG |
| 11 | hsa-miR-598 | 0.15 | MIMAT0003266 | UACGUCAUCGUUGUCAUCGUCA |
| 12 | **hsa-miR-199a-5p** | -0.15 | MIMAT0000231 | CCCAGUGUUCAGACUACCUGUUC |
| 13 | **hsa-miR-204-5p** | -0.14 | MIMAT0000265 | UUCCCUUUGUCAUCCUAUGCCU |
| 14 | hsa-miR-3147 | 0.13 | MIMAT0015019 | GGUUGGGCAGUGAGGAGGGUGUGA |
| 15 | **hsa-miR-1827** | 0.13 | MIMAT0006767 | UGAGGCAGUAGAUUGAAU |
| 16 | **hsa-miR-495** | 0.11 | MIMAT0002817 | AAACAAACAUGGUGCACUUCUU |
| 17 | **hsa-miR-15a-5p** | -0.11 | MIMAT0000068 | UAGCAGCACAUAAUGGUUUGUG |
| 18 | **hsa-let-7g-5p** | -0.10 | MIMAT0000414 | UGAGGUAGUAGUUUGUACAGUU |
| 19 | **hsa-miR-1279** | 0.10 | MIMAT0005937 | UCAUAUUGCUUCUUUCU |
| 20 | **hsa-miR-145-5p** | -0.09 | MIMAT0000437 | GUCCAGUUUUCCCAGGAAUCCCU |
| 21 | hsa-miR-1244 | 0.09 | MIMAT0005896 | AAGUAGUUGGUUUGUAUGAGAUGGUU |
| 22 | hsa-miR-98 | -0.08 | MIMAT0000096 | UGAGGUAGUAAGUUGUAUUGUU |
| 23 | hsa-miR-1322 | 0.08 | MIMAT0005953 | GAUGAUGCUGCUGAUGCUG |
| 24 | **hsa-miR-10b-5p** | -0.07 | MIMAT0000254 | UACCCUGUAGAACCGAAUUUGUG |
| 25 | hsa-miR-1268a | 0.07 | MIMAT0005922 | CGGGCGUGGUGGUGGGGG |
| 26 | **hsa-miR-1246** | -0.06 | MIMAT0005898 | AAUGGAUUUUUGGAGCAGG |
| 27 | hsa-miR-4443 | 0.05 | MIMAT0018961 | UUGGAGGCGUGGGUUUU |
| 28 | hsa-miR-4455 | 0.05 | MIMAT0018977 | AGGGUGUGUGUGUUUUU |
| 29 | **hsa-miR-125b-5p** | -0.03 | MIMAT0000423 | UCCCUGAGACCCUAACUUGUGA |
| 30 | hsa-miR-95 | 0.03 | MIMAT0000094 | UUCAACGGGUAUUUAUUGAGCA |
| 31 | **hsa-miR-195-5p** | 0.02 | MIMAT0000461 | UAGCAGCACAGAAAUAUUGGC |
| 32 | hsa-miR-548ag | 0.02 | MIMAT0018969 | AAAGGUAAUUGUGGUUUCUGC |
| 33 | hsa-miR-24-3p | 0.02 | MIMAT0000080 | UGGCUCAGUUCAGCAGGAACAG |
| 34 | hsa-miR-1290 | 0.02 | MIMAT0005880 | UGGAUUUUUGGAUCAGGGA |
| 35 | hsa-miR-4454 | -0.02 | MIMAT0018976 | GGAUCCGAGUCACGGCACCA |
| 36 | **hsa-miR-891a** | 0.01 | MIMAT0004902 | UGCAACGAACCUGAGCCACUGA |
| 37 | **hsa-miR-199b-5p** | -0.01 | MIMAT0000263 | CCCAGUGUUUAGACUAUCUGUUC |
| 38 | **hsa-miR-494** | 0.01 | MIMAT0002816 | UGAAACAUACACGGGAAACCUC |
| 39 | hsa-miR-1183 | 0.01 | MIMAT0005828 | CACUGUAGGUGAUGGUGAGAGUGGGCA |
| 40 | **hsa-miR-23a-3p** | -0.01 | MIMAT0000078 | AUCACAUUGCCAGGGAUUUCC |
| 41 | hsa-miR-630 | 0.002 | MIMAT0003299 | AGUAUUCUGUACCAGGGAAGGU |

| Supplementary Table 3. IPA significant canonical pathways | | | |
| --- | --- | --- | --- |
| **Ingenuity Canonical Pathways** | **-log(p-value)** | **Ratio** | **Molecules** |
| Glucocorticoid Receptor Signaling | 7.93 | 4.33E-02 | FOS,ICAM1,PIK3R1,FOXO3,TGFB3,KRAS,IL6,CDKN1C,MAPK12,ESR1,MMP1,BCL2 |
| PI3K/AKT Signaling | 7.80 | 6.67E-02 | PPP2R2A,PIK3R1,FOXO3,MDM2,KRAS,CDKN1B,PTEN,MCL1,BCL2 |
| Molecular Mechanisms of Cancer | 7.62 | 3.58E-02 | FOS,BBC3,PIK3R1,DIRAS3,TGFB3,CDK6,KRAS,MDM2,CDKN1B,MAPK12,CDC42,BCL2L11,BCL2 |
| HMGB1 Signaling | 7.48 | 8.25E-02 | FOS,ICAM1,SP1,DIRAS3,PIK3R1,KRAS,CDC42,MAPK12 |
| Pancreatic Adenocarcinoma Signaling | 7.04 | 6.96E-02 | PIK3R1,TGFB3,MDM2,KRAS,CDKN1B,CDC42,MAPK12,BCL2 |
| IL-6 Signaling | 6.73 | 6.56E-02 | COL1A1,FOS,PIK3R1,CRP,KRAS,IL6,MAPK12,MCL1 |
| PTEN Signaling | 6.68 | 6.11E-02 | PIK3R1,FOXO3,KRAS,CDKN1B,CDC42,BCL2L11,PTEN,BCL2 |
| Dendritic Cell Maturation | 6.53 | 4.69E-02 | COL1A2,COL1A1,COL5A3,ICAM1,PIK3R1,IL6,TLR3,MAPK12,COL3A1 |
| TGF-β Signaling | 6.41 | 7.53E-02 | FOS,TGFB3,KRAS,CDC42,MAPK12,ACVR2A,BCL2 |
| Aryl Hydrocarbon Receptor Signaling | 6.28 | 5.67E-02 | FOS,SP1,CDK6,TGFB3,MDM2,IL6,CDKN1B,ESR1 |
| Chronic Myeloid Leukemia Signaling | 6.24 | 6.86E-02 | HDAC4,PIK3R1,CDK6,TGFB3,MDM2,KRAS,CDKN1B |
| Hepatic Fibrosis / Hepatic Stellate Cell Activation | 6.18 | 5.71E-02 | COL1A2,COL1A1,ICAM1,TGFB3,IL6,MMP1,COL3A1,BCL2 |
| Glioblastoma Multiforme Signaling | 5.99 | 5.10E-02 | DIRAS3,PIK3R1,CDK6,MDM2,KRAS,CDKN1B,CDC42,PTEN |
| Role of Tissue Factor in Cancer | 5.79 | 6.42E-02 | PIK3R1,KRAS,RPS6KA5,CDC42,MAPK12,MMP1,PTEN |
| HER-2 Signaling in Breast Cancer | 5.51 | 7.50E-02 | PIK3R1,CDK6,MDM2,KRAS,CDKN1B,CDC42 |
| Atherosclerosis Signaling | 5.46 | 5.34E-02 | COL1A2,COL1A1,COL5A3,ICAM1,IL6,MMP1,COL3A1 |
| Colorectal Cancer Metastasis Signaling | 5.40 | 3.69E-02 | FOS,DIRAS3,PIK3R1,TGFB3,KRAS,IL6,TLR3,MAPK12,MMP1 |
| Prostate Cancer Signaling | 5.38 | 6.59E-02 | PIK3R1,MDM2,KRAS,CDKN1B,PTEN,BCL2 |
| ILK Signaling | 5.27 | 4.32E-02 | FOS,PPP2R2A,DIRAS3,PIK3R1,RPS6KA5,CDC42,MAPK12,PTEN |
| Epithelial Adherens Junction Signaling | 4.94 | 4.86E-02 | TUBB2A,ARPC3,KRAS,CDC42,PTPRM,ACVR2A,PTEN |
| ErbB2-ErbB3 Signaling | 4.93 | 8.62E-02 | SP1,PIK3R1,KRAS,CDKN1B,PTEN |
| Intrinsic Prothrombin Activation Pathway | 4.86 | 1.25E-01 | COL1A2,COL1A1,COL5A3,COL3A1 |
| HGF Signaling | 4.84 | 5.88E-02 | FOS,PIK3R1,KRAS,IL6,CDC42,MAPK12 |
| HIF1α Signaling | 4.82 | 5.88E-02 | PIK3R1,MDM2,KRAS,MAPK7,MAPK12,MMP1 |
| Cholecystokinin/Gastrin-mediated Signaling | 4.79 | 5.94E-02 | FOS,DIRAS3,KRAS,MAPK7,MAPK12,CCKBR |
| Germ Cell-Sertoli Cell Junction Signaling | 4.75 | 4.46E-02 | DIRAS3,PIK3R1,TUBB2A,TGFB3,KRAS,CDC42,MAPK12 |
| Estrogen-Dependent Breast Cancer Signaling | 4.71 | 7.46E-02 | FOS,SP1,PIK3R1,KRAS,ESR1 |
| ERK5 Signaling | 4.68 | 7.81E-02 | FOS,FOXO3,KRAS,RPS6KA5,MAPK7 |

| Supplementary Table 4. IPA significant biologic functions and/or diseases | | |
| --- | --- | --- |
| **Categories** | **P-values** | **Molecules** |
| Cellular Growth and Proliferation | 1.19E-20-1.84E-03 | INSIG1,DIRAS3,PIK3R1,KRAS,IL6,PPM1D,PTEN,LAMC1,BBC3,SOD2,NR1I2,MYBL2,CDKN1C,MCL1,HDAC4,COL4A1,TUBB2A,CDK6,TBK1,MAPK12,KLF4,PTPRM,NASP,TGFB3,FBN1,MAPRE2,MAPK7,ESR1,TCL1A,ICAM1,COL4A2,CDC42,DUSP2,BCL2,DNMT3B,YY1,SP1,FOXO3,TOP2A,KIT,PURA,CAV2,CCKBR,MMP1,TIMP3,CD276,MDM2,PLK1,RPS6KA5,CNOT8,COL1A1,FOS,BMF,SPARC,CDKN1B,ACVR2A,BCL2L11 |
| Cell Death and Survival | 2.44E-17-2.29E-03 | ICAM1,PPP2R2A,PIK3R1,DIRAS3,KRAS,IL6,COL4A2,CDC42,PPM1D,BCL2,PTEN,DNMT3B,YY1,SOD2,BBC3,SP1,GPR37,NR1I2,FOXO3,TOP2A,KIT,MYBL2,TLR3,CDKN1C,MCL1,TIMP3,HDAC4,DDIT4,CDK6,TBK1,PLK1,MDM2,MAPK12,PTPRM,KLF4,COL1A1,FOS,BMF,CRP,TGFB3,SPARC,BNIP3L,CDKN1B,MAPK7,ESR1,ACVR2A,BCL2L11,TCL1A |
| Cell Cycle | 6.48E-16-2.37E-03 | GAS7,PPP2R2A,DIRAS3,KRAS,IL6,CDC42,PPM1D,BCL2,PTEN,DNMT3B,SP1,NR1I2,FOXO3,TOP2A,MYBL2,CDKN1C,DNMT1,HDAC4,PRC1,CDK6,MLF1,MDM2,PLK1,MAPK12,KLF4,COL1A1,FOS,NASP,SPARC,MAPK7,CDKN1B,ESR1 |
| Cancer | 2.03E-14-2.13E-03 | DNMT3A,TRIM9,INSIG1,DIRAS3,PIK3R1,KRAS,IL6,PTEN,PPM1D,SOD2,BBC3,GPR37,NR1I2,MYBL2,CDKN1C,DNMT1,MCL1,HDAC4,COL4A1,DDIT4,TUBB2A,CDK6,KLF4,TGFB3,FBN1,MAPK7,ESR1,COL3A1,ICAM1,TET1,COL4A2,LOXL2,DUSP2,BCL2,COL1A2,YY1,SP1,FOXO3,TOP2A,KIT,NAV3,TLR3,PURA,MMP1,TIMP3,CD276,PRC1,MDM2,PLK1,COL1A1,FOS,CRP,BNIP3L,SPARC,CDKN1B,COL15A1,BCL2L11,ACVR2A |
| Cellular Development | 5.35E-14-2.37E-03 | GAS7,ICAM1,DIRAS3,PIK3R1,KRAS,IL6,CDC42,LOXL2,DUSP2,BCL2,PTEN,DNMT3B,YY1,SOD2,BBC3,SP1,FOXO3,KIT,MYBL2,PURA,CDKN1C,CAV2,MMP1,CCKBR,MCL1,TIMP3,CD276,COL4A1,HDAC4,TUBB2A,CDK6,MLF1,MDM2,PLK1,MAPK12,KLF4,COL1A1,FOS,NASP,BMF,TGFB3,SPARC,CDKN1B,MAPK7,ESR1,ACVR2A,BCL2L11,TCL1A |
| Organismal Injury and Abnormalities | 1.71E-12-2.37E-03 | DNMT3A,TRIM9,PIK3R1,DIRAS3,KRAS,IL6,PTEN,PPM1D,SOD2,NR1I2,MYBL2,DNMT1,MCL1,COL4A1,HDAC4,DDIT4,TUBB2A,CDK6,KLF4,TGFB3,FBN1,MAPK7,ESR1,COL3A1,ICAM1,TET1,COL4A2,DUSP2,BCL2,DNMT3B,COL1A2,YY1,FOXO3,TOP2A,KIT,PURA,CAV2,MMP1,TIMP3,COL5A2,CD276,PRC1,PLK1,MDM2,FOS,COL1A1,COL5A3,CRP,SPARC,BNIP3L,CDKN1B,COL15A1 |
| DNA Replication, Recombination, and Repair | 2.19E-12-2.44E-03 | DNMT3A,PIK3R1,CDK6,MDM2,PLK1,IL6,KLF4,PTEN,BCL2,DNMT3B,FOS,YY1,SOD2,TOP2A,TGFB3,SPARC,MAPK7,CDKN1B,CDKN1C,PURA,ESR1,DNMT1,TCL1A,MCL1 |
| Reproductive System Disease | 2.39E-12-7.71E-04 | ICAM1,TRIM9,DNMT3A,PIK3R1,DIRAS3,TET1,KRAS,IL6,DUSP2,PPM1D,PTEN,BCL2,COL1A2,DNMT3B,YY1,SOD2,NR1I2,FOXO3,TOP2A,KIT,MYBL2,CAV2,PURA,MMP1,DNMT1,MCL1,TIMP3,HDAC4,COL4A1,DDIT4,TUBB2A,PRC1,CDK6,MDM2,PLK1,KLF4,COL1A1,FOS,CRP,FBN1,SPARC,MAPK7,CDKN1B,COL15A1,ESR1,COL3A1 |
| Hematological Disease | 6.2E-12-2.1E-03 | ICAM1,DNMT3A,TET1,KRAS,IL6,COL4A2,CDC42,BCL2,PTEN,SOD2,BBC3,FOXO3,TOP2A,KIT,NAV3,DNMT1,MCL1,HDAC4,COL4A1,TUBB2A,CDK6,MDM2,CRP,CDKN1B,ESR1,BCL2L11,COL3A1 |
| Immunological Disease | 6.2E-12-1.19E-03 | ICAM1,DNMT3A,KRAS,IL6,COL4A2,DUSP2,BCL2,PTEN,BBC3,FOXO3,TOP2A,NAV3,KIT,TLR3,DNMT1,MMP1,MCL1,HDAC4,COL4A1,TUBB2A,CDK6,MDM2,FOS,RERE,CRP,CDKN1B,ESR1,BCL2L11,COL3A1 |
| Connective Tissue Disorders | 6.77E-12-1.22E-03 | ICAM1,COL4A2,IL6,DUSP2,LOXL2,COL1A2,SOD2,NR1I2,FOXO3,KIT,TLR3,PURA,DNMT1,MMP1,MCL1,TIMP3,COL5A2,COL4A1,TUBB2A,CDK6,MDM2,COL1A1,FOS,COL5A3,CRP,SPARC,COL15A1,ESR1,COL3A1 |
| Dermatological Diseases and Conditions | 2.28E-11-1.84E-03 | COL5A2,ICAM1,COL4A1,HDAC4,TUBB2A,KRAS,COL4A2,IL6,BCL2,PTEN,COL1A2,COL1A1,COL5A3,SOD2,TOP2A,TGFB3,NAV3,KIT,COL15A1,ESR1,MMP1,BCL2L11,MCL1,COL3A1 |
| Gastrointestinal Disease | 1.79E-10-2.1E-03 | ICAM1,TRIM9,INSIG1,PIK3R1,TET1,KRAS,IL6,COL4A2,LOXL2,BCL2,PTEN,PPM1D,COL1A2,SOD2,NR1I2,TOP2A,KIT,DNMT1,TIMP3,COL4A1,TUBB2A,PLK1,MDM2,PTPRM,KLF4,COL1A1,FOS,CRP,TGFB3,SPARC,CDKN1B,COL15A1,ESR1,BCL2L11,ACVR2A,COL3A1 |
| Gene Expression | 1.88E-10-5.76E-04 | GAS7,DNMT3A,PIK3R1,DIRAS3,KRAS,IL6,CDC42,LOXL2,PTEN,PPM1D,BCL2,DNMT3B,YY1,SOD2,SP1,NR1I2,FOXO3,MYBL2,TLR3,PURA,CDKN1C,CCKBR,DNMT1,HDAC4,TBK1,RPS6KA5,PLK1,MDM2,MAPK12,KLF4,FOS,TGFB3,CDKN1B,ESR1,TCL1A |
| Respiratory Disease | 6.47E-10-1.49E-03 | TIMP3,TUBB2A,CDK6,MDM2,PLK1,KRAS,IL6,BCL2,PTEN,SOD2,NR1I2,FOXO3,TOP2A,KIT,CDKN1B,CDKN1C,ESR1,MMP1 |
| Tumor Morphology | 1.21E-08-1.24E-03 | CDK6,KRAS,PLK1,IL6,PTEN,BCL2,DNMT3B,COL1A2,COL1A1,FOS,SP1,FOXO3,TGFB3,KIT,CDKN1B,BCL2L11,MCL1 |
| Renal and Urological Disease | 1.64E-08-1.78E-03 | TIMP3,CD276,ICAM1,PIK3R1,TUBB2A,PRC1,MDM2,KRAS,IL6,PTEN,BCL2,FOS,SOD2,NR1I2,CRP,FOXO3,TOP2A,KIT,BNIP3L,ESR1 |
| Tissue Morphology | 1.95E-08-9.95E-04 | TIMP3,ICAM1,PIK3R1,PRC1,FOXO3,KIT,MYBL2,IL6,CDKN1B,ESR1,KLF4,BCL2 |
| Cell Morphology | 2.3E-08-1.49E-03 | TIMP3,ICAM1,KRAS,MDM2,IL6,CDC42,KLF4,BCL2,PTEN,BBC3,SOD2,GPR37,BNIP3L,CDKN1B,CDKN1C,ESR1,BCL2L11,DNMT1,MCL1,COL3A1 |
| Inflammatory Response | 2.35E-08-1.49E-03 | CD276,ICAM1,HDAC4,TUBB2A,MDM2,IL6,DUSP2,PTEN,BCL2,COL1A2,COL1A1,SOD2,FOXO3,CRP,TOP2A,KIT,NAV3,TLR3,ESR1,MMP1,TCL1A,MCL1,COL3A1 |
| Cardiovascular System Development and Function | 2.47E-08-2.29E-03 | TIMP3,ICAM1,COL4A1,PIK3R1,BACE1,KRAS,COL4A2,IL6,CDC42,MAPK12,PTPRM,BCL2,COL1A2,COL1A1,CRP,FOXO3,TGFB3,SPARC,FBN1,CDKN1B,COL3A1 |
| Cellular Movement | 3.73E-08-2.1E-03 | ICAM1,PIK3R1,KRAS,IL6,COL4A2,CDC42,BCL2,PTEN,DNMT3B,YY1,SOD2,SP1,FOXO3,KIT,CCKBR,MMP1,TIMP3,COL4A1,CDK6,MAPK12,KLF4,PTPRM,FOS,COL1A1,CRP,TGFB3,SPARC,MAPK7,CDKN1B,ESR1 |
| Neurological Disease | 3.8E-08-1.52E-03 | ICAM1,PIK3R1,ZFP36L1,IL6,COL4A2,BCL2,PTEN,COL1A2,SOD2,GPR37,NR1I2,TOP2A,NAV3,MMP1,DNMT1,MCL1,TIMP3,HDAC4,COL4A1,TUBB2A,BACE1,MLF1,MDM2,PTPRM,COL1A1,FAM3C,CRP,SPARC,CDKN1B,ESR1,BCL2L11,COL3A1 |
| Endocrine System Disorders | 7.86E-08-2.1E-03 | ICAM1,PIK3R1,DIRAS3,KRAS,IL6,LOXL2,BCL2,PTEN,SOD2,NR1I2,TOP2A,KIT,MYBL2,TLR3,DNMT1,MMP1,TIMP3,COL4A1,TUBB2A,KLF4,PTPRM,FOS,CRP,CDKN1B,COL15A1,ESR1,BCL2L11 |
| Organismal Survival | 9.05E-08-9.05E-08 | SOD2,ICAM1,TOP2A,KIT,KRAS,CDKN1B,IL6,ESR1,BCL2 |
| Cellular Assembly and Organization | 1.53E-07-9.81E-04 | TIMP3,COL5A2,ICAM1,PIK3R1,PRC1,KRAS,MDM2,PLK1,TBK1,IL6,CDC42,KLF4,PTPRM,PTEN,COL1A2,COL1A1,KIT,CAV2,ESR1,COL3A1 |
| Cellular Function and Maintenance | 1.53E-07-1.49E-03 | ICAM1,PIK3R1,KRAS,IL6,CDC42,PTEN,BCL2,COL1A2,SOD2,BBC3,GPR37,KIT,MMP1,CCKBR,MCL1,TIMP3,COL5A2,COL4A1,PRC1,TBK1,PLK1,MDM2,PTPRM,COL1A1,BNIP3L,ESR1,BCL2L11,COL3A1 |
| Psychological Disorders | 2.42E-07-1.52E-03 | TIMP3,ICAM1,PIK3R1,TUBB2A,BACE1,IL6,PTPRM,BCL2,PTEN,FAM3C,SOD2,GPR37,CRP,SPARC,NAV3,CDKN1B,ESR1,BCL2L11,MMP1,DNMT1,COL3A1 |
| Cell-To-Cell Signaling and Interaction | 2.83E-07-2.29E-03 | ICAM1,PIK3R1,IL6,CDC42,BCL2,PTEN,SOD2,NR1I2,FOXO3,TOP2A,KIT,TLR3,CCKBR,MMP1,MCL1,TIMP3,CD276,HDAC4,DDIT4,PTPRM,CRP,SPARC,MAPK7,ESR1,TCL1A |
| Inflammatory Disease | 5.78E-07-1.84E-03 | TIMP3,HDAC4,ICAM1,TUBB2A,CDK6,MDM2,IL6,DUSP2,COL1A2,COL1A1,FOS,SOD2,FOXO3,CRP,TOP2A,NAV3,KIT,TLR3,PURA,MMP1,DNMT1,MCL1,COL3A1 |
